# Dysphagia after Colon Interposition Graft for Esophageal Carcinoma

**DOI:** 10.1155/2012/738205

**Published:** 2012-11-04

**Authors:** C. Spitali, K. De Vogelaere, G. Delvaux

**Affiliations:** Department of Surgery, UZ Brussel, Laarbeeklaan 101, 1090 Jette, Belgium

## Abstract

Colon interposition is an established technique for esophageal reconstruction. We describe the case of primary adenocarcinoma arising in a colonic interposition graft that was performed after total esophagectomy for recurrence adenocarcinoma derived from the Barrett esophagus.

## 1. Introduction

In 1911, Kelling and Vuillet described the anatomic and surgical bases for the use of colon as replacement for the esophagus [1, 2].

Early postoperative complications are common, including necrosis of the transplant, leaks, fistulae, and strictures at the anastomoses. Late complications are rare. We describe an unusual late complication of a primary adenocarcinoma arising in the interposed colon after a right colonic bypass in a patient with adenocarcinoma derived from the Barrett esophagus.

## 2. Case Report

A 66-year-old male presented with history of progressive dysphagia for solid food.

**Figure 1:**
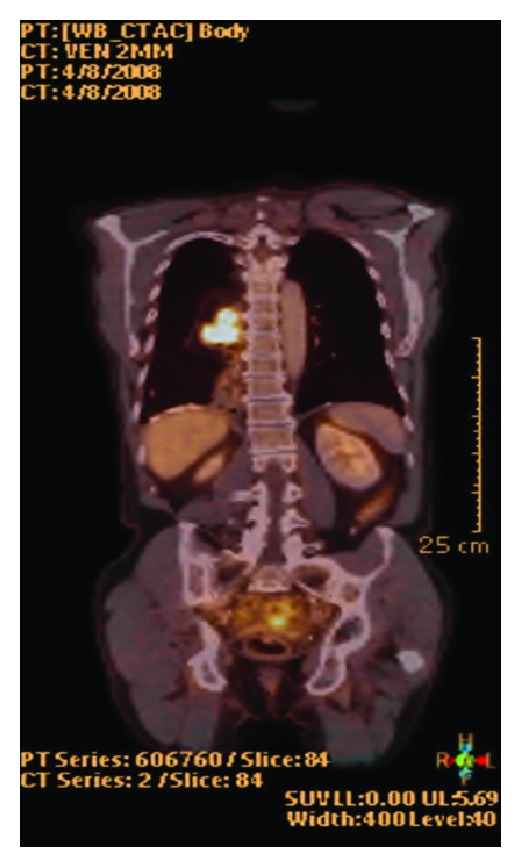
The PET-CT showed a tumor paravertebral right at the level of D7–D9 and no metastatic disease.

**Figure 2:**
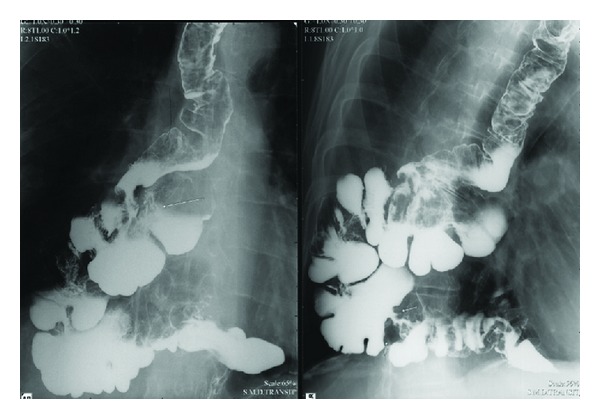
Barium swallow revealed a circumferential mass arising from the colon.

**Figure 3:**
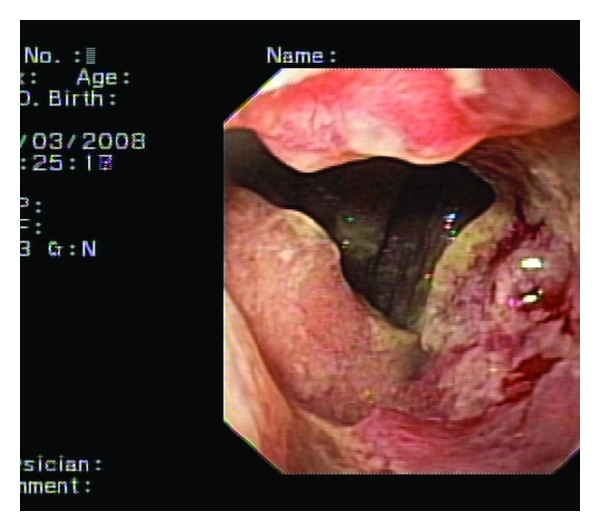
Endoscopy showed circumferential tumor at the ileocaecal junction.

Six years ago, he underwent a proximal gastrectomy and distal esophagectomy for adenocarcinoma derived from Barrett esophagus. Four years later, an esophagoscopy revealed a recurrence at the anastomotic site. A biopsy of the lesion showed a well-differentiated adenocarcinoma of the esophagus. Further investigation showed no evidence of metastatic disease, and a three-stage procedure with total esophagectomy was performed. The right-sided colon from the ileocaecal junction to the mid-transverse colon was used in a prevertebral position to reestablish continuity between the cervical esophagus and the remnant of the pyloric antrum. Histological examination revealed a pT2N0 lesion, and the patient recovered well without major complications. Two years after this intervention, the patient developed a progressive dysphagia for solid food. Endoscopy showed a circumferential mass arising from the colon (Figure 1), and biopsy confirmed a colonic type of adenocarcinoma. Barium swallow revealed the circumferential tumor at the ileocaecal junction (Figure 2). Further investigation with PET scan (Figure 3) demonstrated a tumor paravertebral right at the level of D7–D9 and no metastatic disease. Reintervention was proposed to the patient. Through a right thoracotomy an ileocaecal resection with end-to-end anastomosis was performed with preservation of the “cardial” marginal arcades artery to prevent necrosis of the proximal ileal segment. 

Histological examination revealed a pT2 tumor of the colon and one normal lymphenode. 

The patient recovered well with a good functional conduit and remained disease-free now at 24 months.

## 3. Discussion

The potential use of a pedicled segment of colon to bypass esophageal pathology was first described since the early sixthies. Colonic grafts have been used in the treatment of both benign (e.g., esophageal atresia and stricture) and malignant oesophageal pathology (e.g., cancer of esophagus and cardia). 

Early complications of this procedure are common, including graft necrosis, anastomotic leak, fistulae, and stricture of the anastomosis [3–5]. Other unusual sequelae, including paracolic hiatal herniation and herniation of small intestine through the mesocolon have been reported [6]. 

Late complications are rare: progressive fibrostenosis of the graft, peptic colitis with ulceration of the colonic segment, gastrocolic reflux, and colopericardial and colobronchial fistula were described [4, 7]. The progressive development of diverticular disease in colon interposition has also been reported in previous literature by Nelson and Grayer [8]. 

Primary carcinoma arising in a colonic interposition is obviously rare. Review of literature showed only 11 cases (Table 1). These cases describe the use of colonic grafts in the treatment of both benign (e.g., esophageal atresia and stricture) and malignant oesophageal pathology (e.g., cancer of esophagus and cardia).  Table 2 shows the pathology in the colonic graft at long term and the treatment.

The late development of dysphagia in a patient with a colonic interposition graft should be examined seriously.

Contrast studies of colonic grafts can be difficult to interpret due to altered anatomy. Barium esophagography has the advantage of providing functional evaluation of the graft and integrity of the conduit anastomosis. However, radiographic evaluations and interpretations of the interposed colon may be difficult if there is unfamiliarity with the various surgical procedures and the postoperative appearances [10, 11].

Endoscopy with biopsy should therefore be considered.

Computed tomography plays a limited role in the examination of the interposed colon. It may provide valuable evaluation of the extent of the tumor invasion and for the staging preoperatively.

So far, there is no definable association between the primary carcinoma of the esophagus or stomach and the colon cancer [20, 21].

As more patients are followed over a long period, the later sequelae of colon interposition will become more evident. Whether the interposed segment of colon is more likely to develop carcinoma than a normally sited segment of colon remains to be seen. 

**Figure 4:**
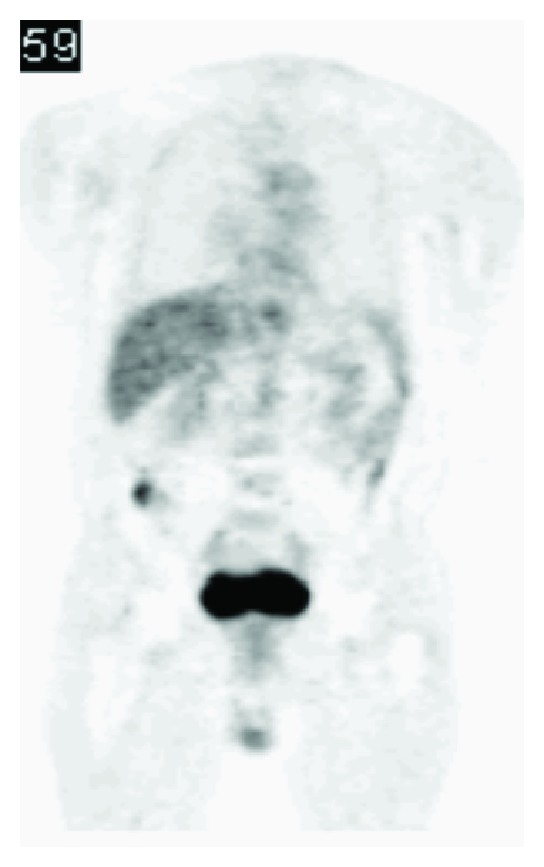
Pre-existing lesion of the colon missed on PET-CT.

In our case, a pre-existing lesion of the colon was indeed missed: during the examinations preoperatively an existing little spot was not remarked on PET scan (Figure 4). This was probably a tubulovillous adenoma of the colon. It is clear that examination of the colon before using it as a graft to exclude colonic disease is preferred.

On the other hand, to rule out the existence of a second primary cancer arising in the interposed colonic mucosa, an endoscopy of the colonic graft should be considered regularly as follow up in the postoperative period.

In summery, malignancy arising in the interposed colon graft is rare. 

Total colonoscopy should be included in the preoperative setting when interposition of colonic segment needs to be used for replacement of the esophagus. This is to detect unexpected lesions.

Development of new symptoms in a patient with a colonic graft should always be taken seriously and investigated.

## Figures and Tables

**Table 1:** Cases of adenoma and adenocarcinoma at the interposed colon segments for esophageal reconstructive surgery reported in the English literature.

Reference	Year of publication	Age (years)	Gender	Original disease and treatment	Time since graft (years)	Pathology
Goldsmith and Beattie [9]	1968	48	Female	Esophageal poorly differentiated epidermoid carcinoma RT 5947 r Right colon for reconstruction	2	Villous adenoma (middle part of graft)
Licata et al. [10]	1978	51	Male	Benign esophageal stricture resulting from ingestion of lye Right colon for reconstruction	11	Adenocarcinoma (middle part of graft)
Haerr et al. [11]	1987	72	Male	SCC at the junction of mid and lower third of esophagus RT 46 Gray in 23 fractions Right colon for reconstruction	9	Adenocarcinoma (colonogastric junction)
Houghton et al. [12]	1989	64	Male	Benign esophageal stricture Right colon for reconstruction	20	Villous adenoma (esophagocolonic junction)
Lee et al. [13]	1994	67	Male	Advanced adenocarcinoma of EG junction Jejunal graft initially with graft necrosis Right colon graft at subcutaneous rout 1 year later	14	Adenocarcinoma (middel part of graft)
Theile et al. [14]	1991	75	Female	Postcricoid SCC Pharyngolaryngectomy with right colon reconstruction	20	Adenocarcinoma (esophagocolonic junction)
Altorjay et al. [15]	1995	65	Male	Esophageal stricture distal third Lower third resection of esophagus Left colon for reconstruction	5	Adenocarcinoma (middle part of graft)
Goyal et al. [16]	2000	78	Male	Gastric cardiac carcinoma Distal esophagectomy, partial gastrectomy with tube reconstruction Gastric tube and oesophagus avascular necrosis complicated Total gastrectomy, subtotal esophagectomy with right colon for reconstruction	7	Adenocarcinoma (middle part of graft)
Liau et al. [17]	2004	79	Male	Esophageal cancer	30	Primary adenocarcinoma (middel third of graft)
Hsieh et al. [18]	2005	57	Male	Alkaline corrosive injury of the esophagus	37	Primary adenocarcinoma (anastomotic site)
Roos et al. [19]	2007	79	Male	Esophageal adenocarcinoma	7	Primary adenocarcinoma (colonogastric junction)
Spitali	2012	60	Male	Esophageal adenocarcinoma (malignant degeneration of Barrett's esophagus	6	Primary adenocarcinoma (anastomotic site)

**Table 2:** Treatment of adenoma and adenocarcinoma at the interposed colon segments for esophageal reconstructive surgery.

Reference	Year of publication	Pathology	Treatment
Goldsmith and Beattie [9]	1968	Villous adenoma (middle part of graft)	Segmental resection distal colon bypass and cologastrostomy
Licata et al. [10]	1978	Adenocarcinoma (middel part of graft)	Not specified
Haerr et al. [11]	1987	Adenocarcinoma (colonogastric junction)	Radio and chemotherapy because of tumor unresectable (invasion of sternum and mediastinum)
Houghton et al. [12]	1989	Villous adenoma (esophagocolonic junction)	Resection colonic interposition and gastric interposition
Lee et al. [13]	1994	Adenocarcinoma (middel part of graft)	Resection lower part colonic graft and reanastomosis with jejunum
Theile et al. [14]	1991	Adenocarcinom (esophagocolonic junction)	Resection upper part colonic graft and free jejunal graft
Altorjay et al. [15]	1995	Adenocarcinoma (middle part of graft)	Resection interposed colon and Roux-en-Y esophagojejunostomy
Goyal et al. [16]	2000	Adenocarcinoma (middle part of graft)	
Liau et al. [17]	2004	Primary adenocarcinoma (middel third of graft)	Chemotherapy
Hsieh et al. [18]	2005	Primary adenocarcinoma (anastomotic site)	Resection whole colonic graft, cervical esophagostomy, and feeding gastrostomy
Roos et al. [19]	2007	Primary adenocarcinoma (colonogastric junction)	Resection colon graft, cervical oesophagostomy, and feeding jejunostomy
Spitali	2012	Primary adenocarcinoma (anastomotic site)	Ileocecal resection and end-to-end anastomosis
